# To Revascularise or Not To Revascularise, That Is the Question: the Diagnostic and Management Conundrum of Ischaemic Cardiomyopathy

**DOI:** 10.1007/s11886-016-0726-9

**Published:** 2016-04-26

**Authors:** Natalia Briceno, Divaka Perera

**Affiliations:** British Heart Foundation Centre of Excellence and National Institute for Health Research Biomedical Research Centre, Cardiovascular Division, St Thomas’ Hospital Campus, Kings College London, London, UK

**Keywords:** Ischaemic cardiomyopathy, Viability, Ischaemia, Revascularisation

## Abstract

Ischaemic cardiomyopathy is an important cardiovascular condition that has differing pathophysiological substrates and clinical manifestations. Contemporary management involves the administration of heart failure pharmacotherapy and device therapy where indicated, which has good prognostic data to support it. Whilst the role of revascularisation is clear in those patients presenting with an acute coronary syndrome or angina, the role in those patients presenting either incidentally, with predominant heart failure symptoms, or in those presenting with acute heart failure with an associated elevated troponin is less well defined and lacks randomised outcome data to support its adoption. The aim of this review is therefore to discuss the challenges in the diagnosis of ischaemic cardiomyopathy with a review of the existing imaging modalities that can facilitate, and to revisit the variety of clinical presentations that can occur, with particular emphasis on the contemporary role of revascularisation in these cohorts of patients.

## Introduction

Heart failure secondary to coronary artery disease, also known as ischaemic cardiomyopathy (ICM), is an important cardiovascular condition that has a significant impact on both morbidity and mortality [1, 2] that is managed across different disciplines within the field of cardiology. ICM is increasing in incidence [3] due to technological advancements in interventional and pharmacotherapy for acute myocardial infarction with overall improved survival, with a resultant increase in the development of persistent left ventricular systolic dysfunction (LVSD) in a subset of patients. It is important to note that ICM rather than being a single entity represents a spectrum of differing pathophysiological processes and clinical manifestations. Clinically, patients can present with stable angina, acute coronary syndromes and heart failure (with a varying spectrum of severity) and can even present incidentally without overt symptoms. The underlying pathophysiology is more complex, and multiple mechanisms can exist in one patient. These include myocardial stunning, hibernation and scar, leading to increased left ventricular volumes (with irreversible remodelling) and varying degrees of regional or global hypocontractility.

Evidence-based heart failure pharmacotherapy and device therapy have been the mainstay of treatment for patients with ICM across these differing pathophysiological and clinical substrates, with good prognostic data to support it [4–7]. Whilst the role of revascularisation is well defined and recommended in patients with an acute coronary syndrome (ACS) and significant angina in several international guidelines [8, 9–11], this is less clear in those patients with predominant heart failure symptoms or those who are asymptomatic. The potential theoretical benefits of revascularisation are often weighed up against its potential risks, particularly in those patients where the evidence base is less well established. This has been further debated following the publication of the Surgical Treatment for Ischaemic Heart Failure (STICH) trial, which looked at the role of surgical revascularisation in ICM and did not meet its primary outcome of all-cause mortality [12••]. These results have led to a lack of clear consensus on how these patients should be managed with regards to revascularisation. The aim is therefore to review the issues surrounding the diagnosis of ICM and its varying clinical spectrum, and the role of revascularisation in the management of the subset of patients who do not have angina as their predominant symptom or who have not presented with an acute myocardial infarction.

## The Diagnostic Conundrum

ICM classically has been considered as a syndrome of heart failure that is secondary to coronary artery disease. The term cardiomyopathy, however, can be misleading and is often contested by heart failure physicians, as cardiomyopathy relates more to a primary myopathic process. It is important to distinguish ICM from other causes of a cardiomyopathy, particularly for prognostic purposes as these patients have been shown to have increased rates of mortality compared with those with a non-ischaemic aetiology [13–15]. Felker et al. sought to develop a prognostically powerful universal definition of ICM [16], through a retrospective observational study of 1921 heart failure patients. Their results mirrored previous studies, indicating a worse mortality with an ischaemic aetiology; however, they also found that more extensive coronary artery disease was independently associated with worse survival. Interestingly, they also identified a group of patients that had single-vessel coronary artery disease (excluding the left main stem and proximal left anterior descending artery), that using classical definitions would have been classified as having ICM, yet they had similar outcomes to those patients with no coronary artery disease. By reclassifying these patients into the non-ischaemic group, the investigators were able to add power to their binary classification model for diagnosis. In a real-world setting, however, it is often left to the judgement of the treating physician to decide whether the coronary disease identified is out of proportion to the extent of myocardial dysfunction. Techniques to further differentiate the true ‘ischaemics’, with the ‘non-ischaemics’ are warranted, and data is needed on the clinical progression and management of those patients with mixed aetiology.

### The Role of Invasive and Non-invasive Imaging in the Diagnosis of ICM

Invasive coronary angiography remains the gold standard in diagnosing coronary artery disease, and it is currently recommended in patients with heart failure and anginal symptoms or in those patients with demonstrable ischaemia and viability but without angina [9, 11]. Even though to date there is no trial data to support revascularisation in all patients with ICM, the demonstration of coronary artery disease can be important for both future management and prognostic purposes. The presence of coronary disease will dictate important evidence-based pharmacotherapy, including aspirin and statins, which play an important role in preventing ischaemic events, which if they occur can worsen LV function and have a significant impact on morbidity and mortality. It is also important to note that not only the presence but also the severity of coronary artery disease is of prognostic significance and may in the future influence the decision to revascularise or not if the evidence base supports it. This was recently evaluated by a substudy of the STICH trial, where the investigators studied the impact of coronary anatomy and LV dimensions (three-vessel coronary artery disease, ejection fraction below 28 %, and end-systolic volume index above 79 ml/m^2^) on the effect of coronary artery bypass grafting (CABG) in ICM [17••]. The investigators found that patients with more severe coronary artery disease, worse remodelling and lower ejection fraction received a significant mortality benefit from surgical revascularisation. In those patients randomised to medical therapy, there was significantly higher mortality in patients with two or three of the prespecified prognostic factors (*p* < 0.001). However, invasive coronary angiography is not without its risks and is therefore not recommended as a blanket investigation for all patients with heart failure. CT angiography may be a less invasive alternative in these patients, with the added benefits of demonstrating regional wall thickness and with certain imaging sequences detecting the presence or absence of regional wall motion abnormalities.

Viability testing also plays an important role in the diagnostic work-up of ICM. Viability is a term that is used to describe, on the basis of varying imaging features, regions of the myocardium that have the potential to improve following revascularisation. These regions of the myocardium, despite being hypocontractile, demonstrate contractile reserve and have preserved metabolic activity. Imaging plays an important role in distinguishing areas of the myocardium that are scarred and have undergone irreversible remodelling to those areas that are in a reversible state of hypocontractility, as an adaptation to reduced myocardial blood flow. The different imaging modalities that are available target different pathophysiological mechanisms that identify areas that are viable. Whilst echocardiography is the most widely available modality in assessing viability, it is the least sensitive and specific due to its low spatial resolution and the predominant dependence on qualitative measures. Dobutamine stress echocardiography (DSE) involves the administration of dobutamine at low, intermediate and high doses to assess the contractile reserve of myocardial segments. A biphasic response has been shown to be a hallmark of viability, with an initial improvement in contractility with low dose (demonstrating contractile reserve), with a subsequent deterioration in contractility at high dose indicating ischaemia [18]. It is important to note that whilst the presence of both viability and ischaemia have been shown to be predictive of LV recovery following revascularisation, the addition of ischaemia testing can aid the diagnosis of ICM particularly in patients where the significance of epicardial disease is unclear. Nuclear modalities such as positron emission tomography (PET) and single-photon emission computed tomography (SPECT) provide information regarding myocardial perfusion and metabolism, with PET being one of the most sensitive in identifying viable myocardium (albeit at lower specificity than other modalities) [19]. The main hallmark of viable myocardium is the presence of perfusion metabolic mismatch (reduced perfusion with persistent metabolic activity). Several studies have shown its clinical utility in predicting recovery following revascularisation [20–23], but its reduced availability and cost have limited its widespread use. Cardiac magnetic resonance (CMR) imaging is emerging as the one imaging modality that can provide information on contractile reserve (low-dose dobutamine with cine imaging), scar (late gadolinium enhancement, LGE) and perfusion. LGE on CMR demonstrates areas of the myocardium that consist of fibrosis. It has been shown that areas of hypocontractility without scar are highly likely to recover function following revascularisation; conversely, areas with greater than 50 % of transmurality of LGE have a small chance of recovery following revascularisation [24]. In patients with areas of scar less than 50 %, the addition of a low-dose dobutamine run with cine imaging can highlight those areas that have contractile reserve [25]. Despite decades of observational studies demonstrating the relationship between the presence of viability using various non-invasive imaging techniques and the improvement in mortality in patients revascularised [19], this has not been borne in randomised trials [26, 27], suggesting that there are other factors at play other than viability that predict myocardial recovery and a mortality reduction.

## ICM Presenting as Acute Heart Failure with an Elevated Troponin

Acute heart failure is a common and well-recognised complication of ischaemic cardiomyopathy. This can be in the context of a de novo presentation with acute heart failure (in a patient with no prior history) or acute decompensation in a patient with chronic heart failure. Most of these patients will be managed in a similar manner in most cardiology units around the world, with a combination of intravenous diuretics, nitroglycerin, morphine, non-invasive ventilation and, where necessary, mechanical circulatory support. However, the challenge is in deciding which patients should be put forward for angiography and acute revascularisation. The FINN-AKVA study group sought to identify patient characteristics, varying management strategies and survival in patients with acute heart failure with and without an ACS [28]. They found that patients with an ACS present more often with de novo heart failure and had a more severe clinical manifestation of acute heart failure. Short-term mortality was higher, and length of hospital stay was longer in those patients with concurrent ACS. It is therefore important to distinguish which patients have a concurrent ACS and, therefore, should be put forward for coronary angiography with subsequent revascularisation in order to optimise their outcomes. Most patients presenting with acute heart failure have an elevation of their troponin. Aside from those presenting with acute chest pain or ECG changes suggestive of myocardial ischaemia where a diagnosis of ACS is clear, those patients presenting with acute heart failure and a mild to moderate rise in their troponin represent a big management challenge. A recent study evaluated the value of high-sensitivity troponin for identifying high-risk patients with acute heart failure [29]. They found that 93 % of patients enrolled had a high-sensitivity troponin above the normal value. Patients with worse LV dysfunction had higher troponin values; however, on multivariate analysis, only the need for inotropes predicted increased mortality. An analysis of the RELAX-AHF study in a much larger cohort of patients found that patients with a higher troponin value also had higher N-terminal pro-brain natriuretic peptide levels, suggesting a pathophysiological link between myocardial necrosis and acute LV haemodynamic changes [30•]. They found that higher baseline troponin values were associated with worse overall outcomes. Another study by Latini et al. looked at baseline troponin T levels in the Valsartan in Heart Failure Trial (Val-HeFT) population, which consisted of a group of stable heart failure patients. They found that troponin T was detected using the high-sensitivity assay in 92 % of the patients enrolled. They found that patients with elevated troponin T concentrations (with the standard assay) or elevated concentrations above 0.012 ng/mL using the high-sensitivity assay were the greatest predictor for mortality and heart failure hospitalisation [31]. Early revascularisation has been shown to reduce mortality in those patients presenting with elevated troponin levels [32], but the level of troponin at which this benefit becomes apparent has yet to be elucidated. The release of brain natriuretic peptide during an acute myocardial infarction has also been shown to be predictive of outcome [33], but like troponin, its impact on guiding management has not been tested in a randomised fashion.

When faced with a patient presenting with acute heart failure in the context of ICM, a decision about whether or not to take the patient to the catheter laboratory should be made early. Patients with either moderate or high levels of troponin should be considered for angiography. Once coronary anatomy is delineated, a Heart Team approach should be adopted, with the addition of further viability studies if required, to decide whether revascularisation should be undertaken and through which means. This should be considered carefully, as these patients who have potentially the most to gain are also at the highest risk of events during revascularisation [34, 35]. In those patients with only minor elevations of troponin levels, other factors such as clinical presentation, renal function, baseline troponin levels if known and electrocardiographic features should be considered, alongside viability testing.

## ICM Presenting with Predominant Heart Failure Symptoms

Despite decades of observational data showing a benefit with revascularisation in patients with LVSD, particularly in those with myocardial viability [19], there is no randomised trial data to date that supports revascularisation in ICM for prognosis. As a result, there are conflicting guidelines on how these patients should be managed. The search for hibernating myocardium (through viability testing prior to revascularisation) has been the main clinical and research focus in patients with ICM for many years. Rahimtoola was one of the first to use the term hibernation to describe a hypocontractile myocardium that is in a down-regulated state as a result of processes occurring at both the molecular and cell levels that improves contractility following revascularisation [36]. This interest in ICM and hibernation stemmed from the publication of the landmark surgical revascularisation studies, but translation of this data to current practice is limited by the selection bias inherent in most observational studies, out-dated pharmacotherapy for heart failure used and the fact that most patients undergoing revascularisation of ICM had predominant anginal symptoms [37–39].

### Evidence for Revascularisation

There is a large body of observational data that suggests a mortality benefit with revascularisation in patients with ICM. A meta-analysis performed by Allman et al. included 24 observational studies that assessed viability with a variety of different imaging modalities [19]. A total of 3088 patients were included. When groups were stratified according to those revascularised and those that were not, there was a significant difference in mortality, with a much lower mortality rate observed in those revascularised. There was also a significant improvement in mortality in those treated with revascularisation that had significant viability. However, observational data is well known to be inherent to many biases, particularly selection, which substantially limits what can be concluded from the results.

The only two published randomised trials to date were neutral in terms of their primary outcomes. The HEART-UK trial was a multi-centre RCT that sought to answer the question whether a strategy of invasive coronary angiography and where suitable, revascularisation improved mortality in patients with demonstrable viability [40]. Unfortunately, this trial was terminated early due to slow recruitment and hence was underpowered to demonstrate any mortality differences. It was also noted that a large proportion of patients did not undergo their assigned intervention. The STICH trial [12••], which is the largest completed trial to date that assessed the impact of surgical revascularisation on clinical outcomes in ICM, did not demonstrate an overall mortality benefit with revascularisation. One thousand two hundred twelve patients with an ejection fraction of less than 35 % and coronary artery disease were randomly assigned to undergo CABG alongside optimal medical therapy (OMT) or OMT alone. Forty-one per cent of patients in the OMT group reached the primary end point of all-cause mortality, compared with 36 % of patients in the CABG group (*p* = 0.12). There were some signals of benefit seen in the secondary endpoints, with a trend towards a reduction in cardiovascular cause of death in the CABG group (*p* = 0.05). When the mode of death was investigated, CABG was found to significantly reduce sudden death and fatal pump failure deaths (hazard ratios 0.73 and 0.64, respectively) [41]. There was also a significant reduction in deaths due to myocardial infarctions. These effects were principally seen late following CABG; in the first 30 days, there was excess mortality in the surgical group. It is important to note that approximately 10 % of patients assigned to each arm crossed over into the other arm. In an as-treated analysis, CABG did result in a significant reduction in the primary end point [42].

Six hundred and one out of the 1212 enrolled into the STICH trial underwent viability assessment. This was at the discretion of the recruiting clinicians and was performed in a non-randomised fashion. A subgroup analysis of these patients was performed by Bonow et al., and a total of 487 patients were found to have viable myocardium as per prespecified definitions [27] on the basis of SPECT and dobutamine stress echocardiography (DSE) imaging. In these patients, revascularisation was not associated with a reduction in all-cause mortality. One of the main limitations of this substudy, apart from the fact that viability testing was not mandated in the overall STICH protocol, is that it lacked power to assess the impact of viability, particularly as only 60 patients without viability underwent CABG. The STICH investigators have attempted to find other factors that could predict benefit of revascularisation. A subgroup analysis of patients with angina in the STICH trial was performed, and no association between the presence of angina and increased survival benefit from surgical revascularisation was found in an intention-to-treat analysis [43]. This is in contrast to previous studies looking at the impact of symptoms on prognosis in ICM.

As with many other randomised trials that involve patients who are difficult to enrol, more hypotheses have been generated and there were more questions asked than answers delivered. The results of the STICH trial certainly challenge the long-held clinical dogma, although the latter are based on observational studies which themselves have many limitations. There is clearly a need to perform more robust randomised studies that would refute or clarify the questions left in the wake of the STICH trial. Until then, patients should continue to be managed on an individual basis. Certainly, at present, we do not have the evidence to mandate revascularisation in all patients with ICM, neither do we have evidence to mandate viability testing in all patients with ICM. The multi-centre open-labelled REVascularisation for Ischaemic Ventricular Dysfunction-British Cardiovascular Intervention Society-2 (REVIVED-BCIS-2, NCT01920048) trial [45] is currently recruiting patients in the UK, which will investigate the effect of percutaneous coronary intervention in patients with ICM and evidence of viability. The Alternative Imaging Modalities in Ischaemic Heart Failure (AIMI-HF) trial is also recruiting, which will compare the effect of standard imaging modalities (single-photon emission computed tomography) to advanced imaging modalities (cardiac magnetic resonance imaging or positron emission tomography) on the composite clinical end point of cardiac death, myocardial infarction, resuscitated cardiac arrest and cardiac rehospitalisation [46]. The results of these and future randomised studies are eagerly awaited.

At present, existing international guidelines recommend revascularisation in patients with LV dysfunction with coronary anatomy that is suitable for revascularisation for prognostic purposes [8, 9, 11, 44]. The most recent European Society of Cardiology (ESC) guidelines on myocardial revascularisation recommend CABG in patients with LV systolic dysfunction and left main stem disease with class I level evidence C [8]. PCI may be considered if anatomy is suitable in the presence of viable myocardium and when surgery is not indicated (class IIb level evidence C). Revascularisation in stable coronary artery disease in patients with a LVEF less that 40 % is given class I level evidence A for prognostic purposes. The 2013 American Heart Association heart failure guidelines recommend that CABG should be undertaken in patients with operable anatomy and ICM, with class IIb level evidence B, irrespective of the presence of viability [9]. These guidelines are based predominantly on the results of observational studies and the STICH results, the latter of which did not meet its primary outcome. Please see Table 1 for a summary of the current international guidelines.

**Table 1:** Summary of existing international guidelines on revascularisation in patients with LV systolic dysfunction with predominant heart failure symptoms

Society	Guideline	Year	Recommendation	Class	Level
AHA	CABG	2011	CABG to improve survival is reasonable in patients with mild to moderate LV systolic function (EF 35–50 %) and significant (≥75 % diameter stenosis) multi-vessel CAD or proximal LAD stenosis, where viable myocardium is present in the region of intended revascularisation	IIa	B
AHA	CABG	2011	CABG might be considered with the primary or sole intent of improving survival in patients with stable IHD with severe systolic dysfunction whether or not viable myocardium is present	IIb	B
AHA	Heart failure	2013	CABG should be considered in patients with ICM and operable coronary anatomy whether or not viable myocardium is present	IIb	B
ESC	Heart failure	2012	CABG is recommended for patients with angina and significant left main stem stenosis, who are otherwise suitable for surgery to reduce the risk of premature death	I	C
ESC	Heart failure	2012	PCI may be considered as an alternative to CABG in patients unsuitable for surgery	IIb	C
ESC	Myocardial revascularisation	2014	Revascularisation for prognosis in patients with 2- or 3-vessel coronary artery disease with stenosis >50 % and impaired LV function (EF <40 %)	I	A
ESC	Myocardial revascularisation	2014	CABG is recommended in left main stem stenosis in patients with severe LV dysfunction	I	C

## ICM in the Asymptomatic Patient

Little is known about the incidence and clinical course of patients with asymptomatic ischaemic cardiomyopathy. Data from heart failure trials and observational studies indicate that this population is not insignificant and are at significant risk of development of heart failure symptoms, heart failure hospitalisation and mortality [47, 48]. These patients often present incidentally following an abnormal ECG, often performed for an unrelated reason. Whilst angiography is very useful to identify the cause of LV dysfunction and aid further pharmacotherapy to target coronary artery disease, the decision to revascularise like in patients with predominant heart failure symptoms is complex. As with all other presentations of ICM, good heart failure therapy and consideration of device therapy is mandated. In terms of the diagnostic and management work-up, viability testing should be considered alongside testing for ischaemia and a Heart Team approach should be adopted. Panza et al. identified the patients out of the STICH population that underwent either a radionuclide stress test or a DSE [49], and sought to assess the prognostic significance of stress-induced ischaemia. There was no difference in the mortality between patients with or without ischaemia (*p* = 0.657). When the patients were stratified according to their treatment, no interaction was observed between the treatment effects and the presence or absence of ischaemia. This suggests that the presence of ischaemia does not predict which patients would benefit from revascularisation in this cohort of patients. This is in contrast with many previous studies indicating a survival benefit with revascularisation in those patients with significant ischaemia; however, the majority of these studies did not enrol patients with a reduced ejection fraction. A large observational series by Hachamovitch et al. in a group of patients undergoing stress-rest myocardial perfusion scintigraphy found that although the magnitude of ischaemic myocardium is associated with a survival benefit with revascularisation, this was only seen in patients without a prior myocardial infarction [50]. Until further studies are performed in this particular cohort of patients, revascularisation should not be recommended for all patients, and the main focus should be on managing their LV dysfunction.

## Conclusions

ICM provides a fascinating clinical conundrum, relating to the vast spectrum of underlying pathophysiological states and clinical manifestations. Not only is the diagnosis a challenge, but also the appropriate treatment especially with respect to revascularisation is not fully known. Whilst the mainstay of evidence-based treatment of ICM should focus on pharmacotherapy for treating coronary artery disease and heart failure and on device therapy where indicated, the role of revascularisation needs to be elucidated further, with the final aim of developing individualised therapy that takes into account different factors that may predict benefit from revascularisation. This includes severity of LV dysfunction, severity of coronary artery disease, evidence of viability and presence or absence of ischaemia, which all need to be tested prospectively, with the final aim of developing a model that can guide the Heart Team in the management of this complex and high-risk group of patients to ultimately improve outcomes.
